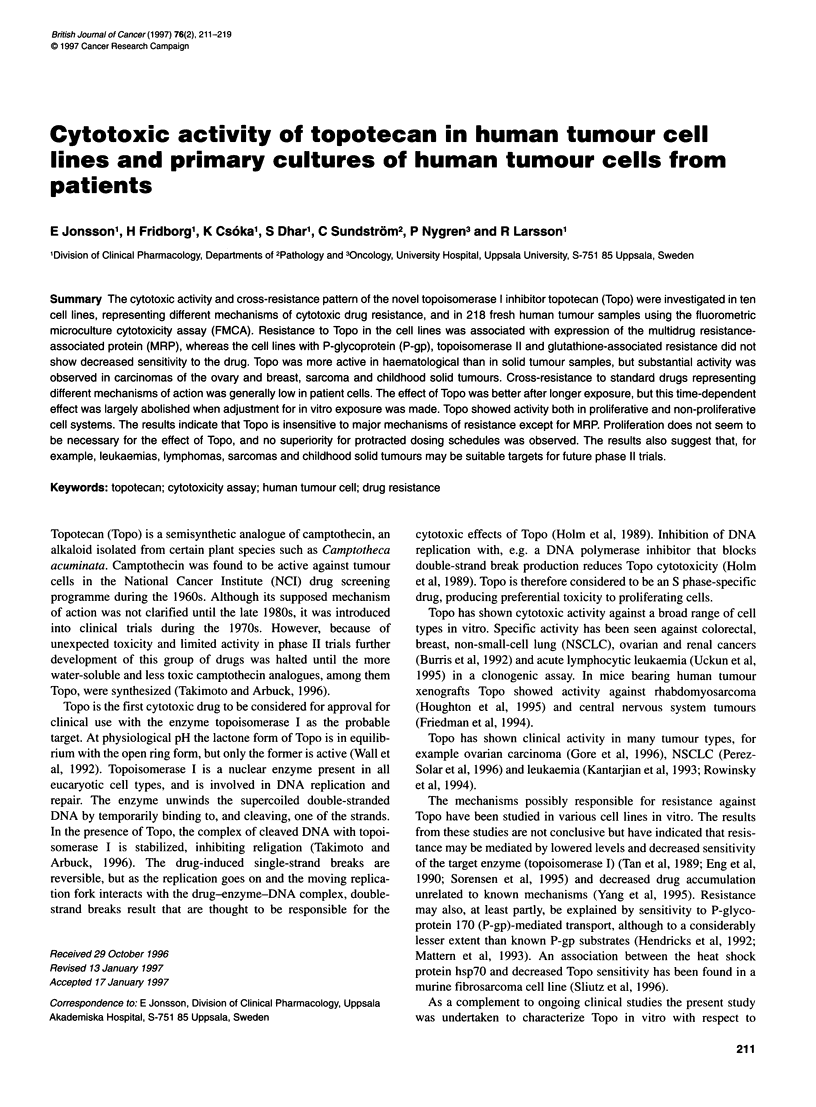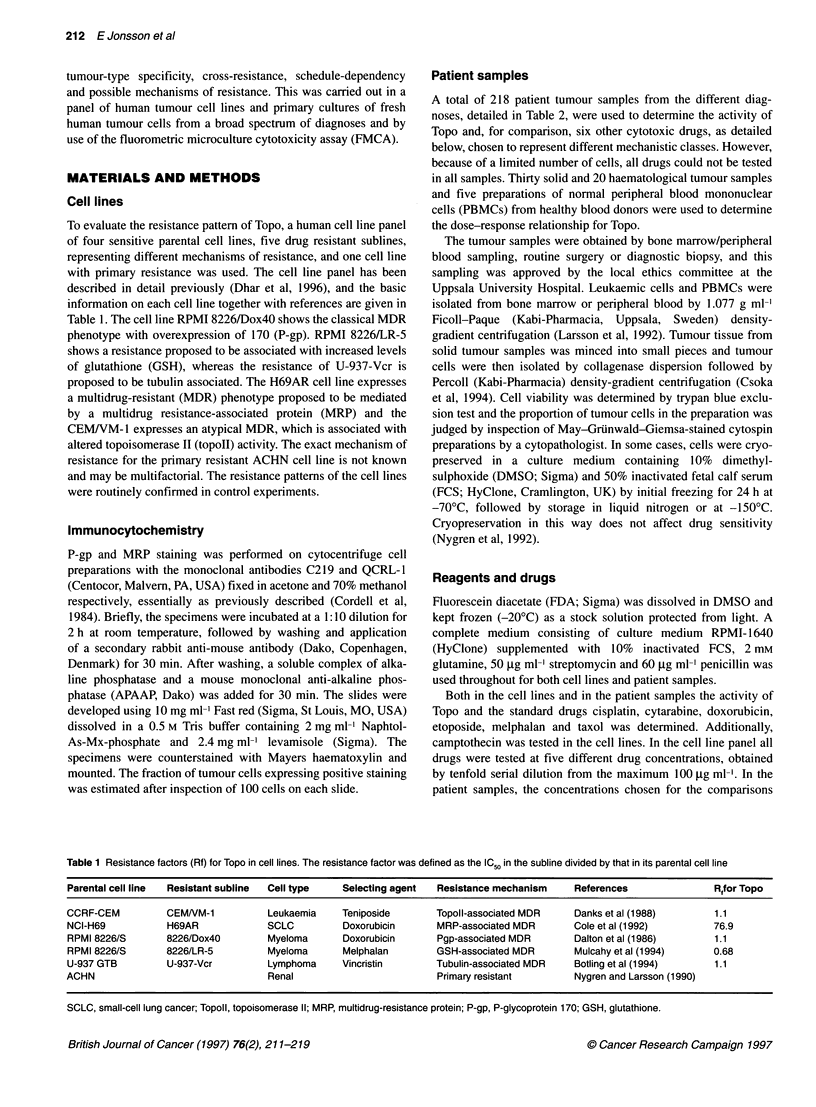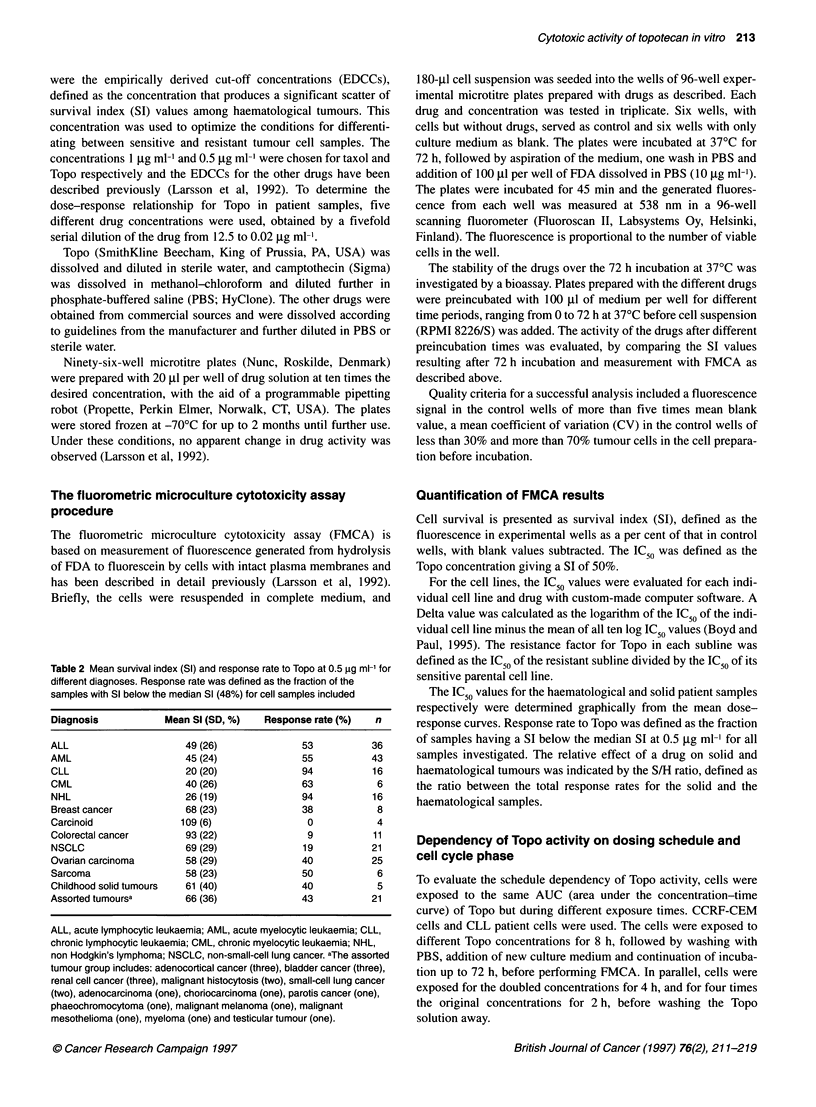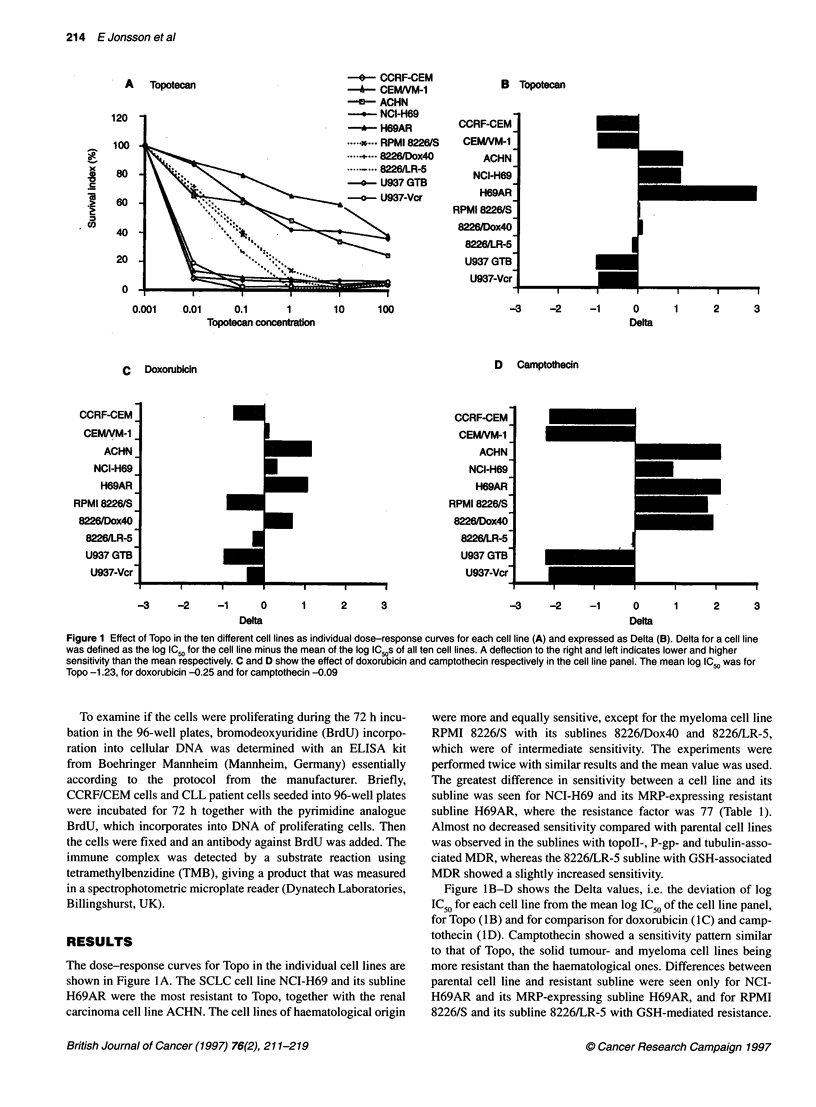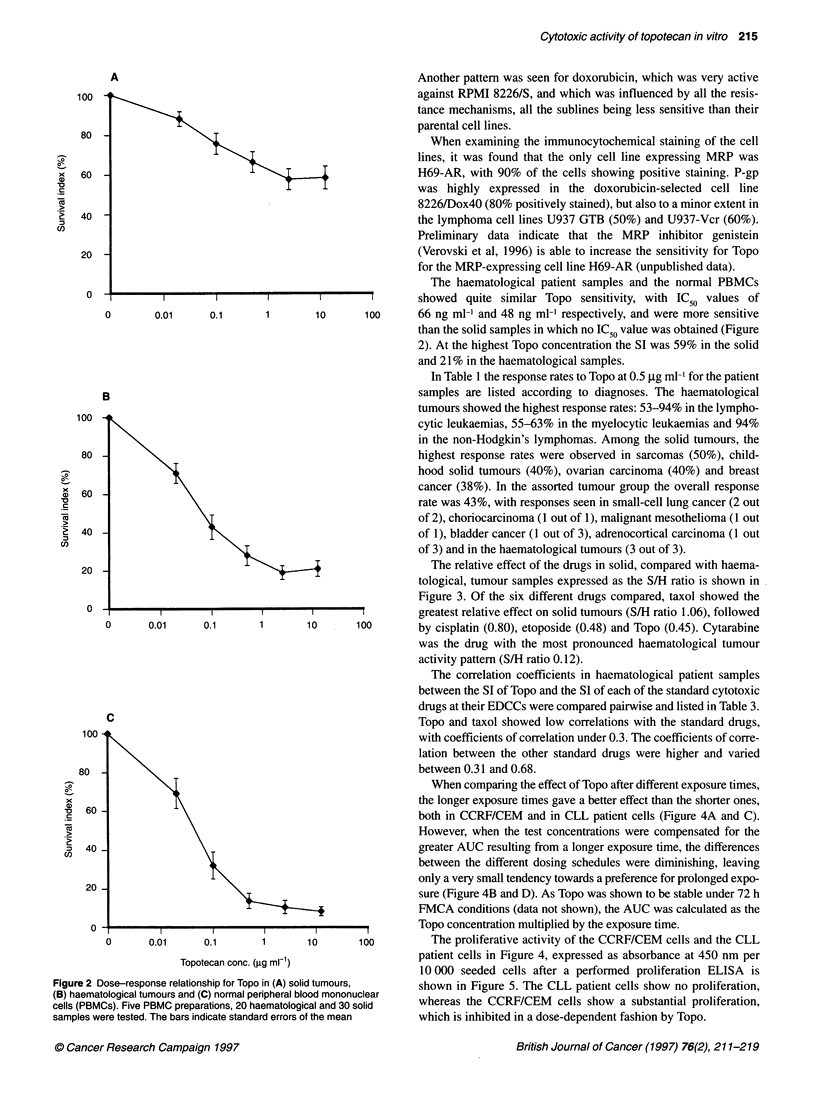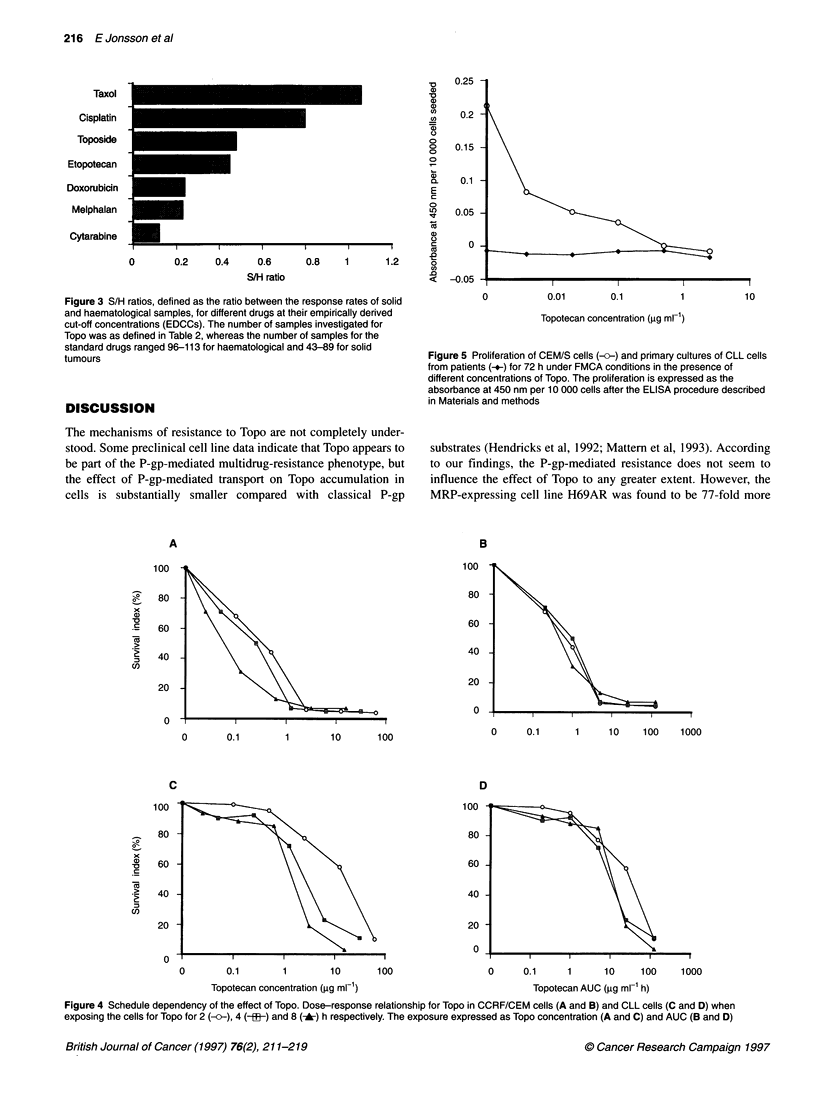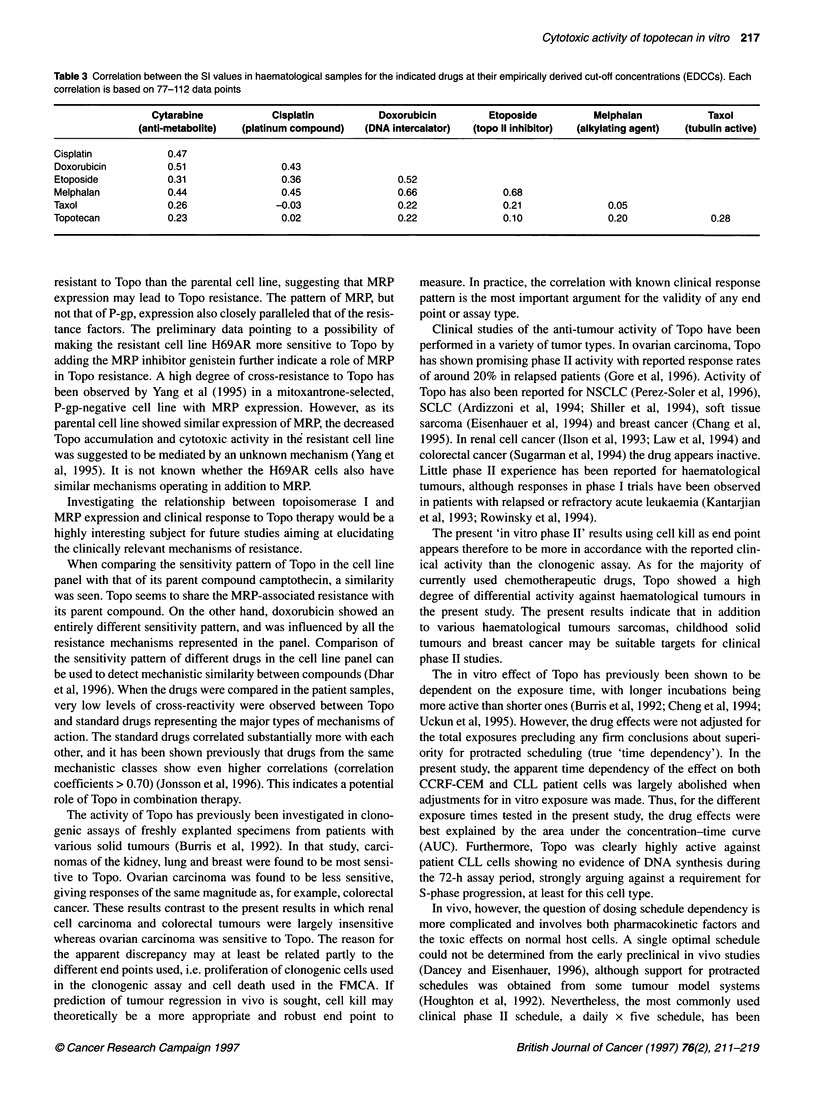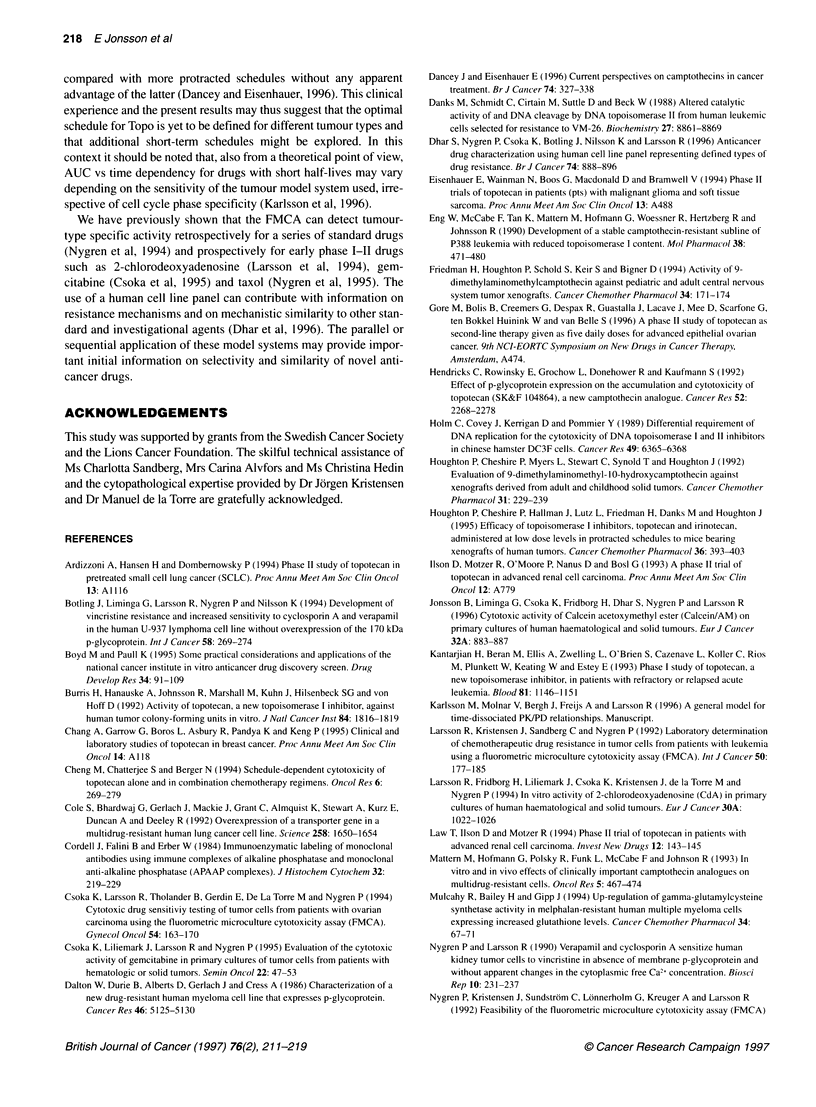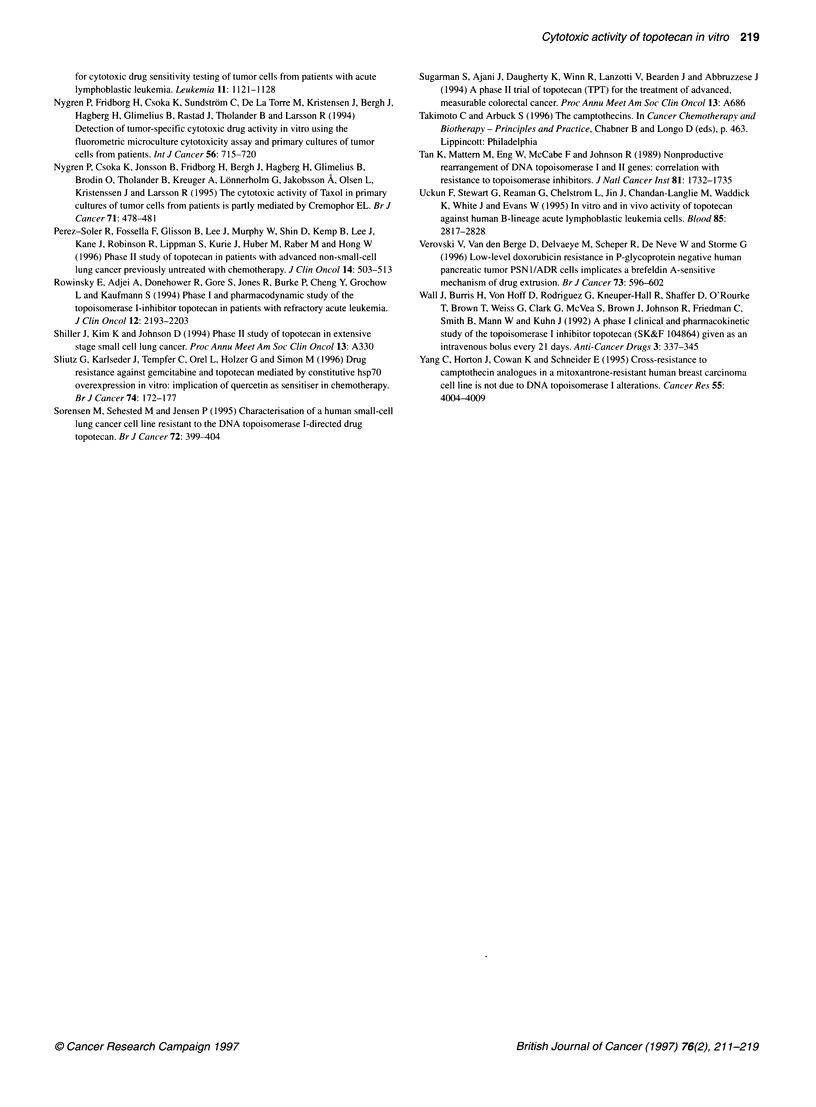# Cytotoxic activity of topotecan in human tumour cell lines and primary cultures of human tumour cells from patients.

**DOI:** 10.1038/bjc.1997.364

**Published:** 1997

**Authors:** E. Jonsson, H. Fridborg, K. CsÃ³ka, S. Dhar, C. SundstrÃ¶m, P. Nygren, R. Larsson

**Affiliations:** Division of Clinical Pharmacology, University Hospital, Uppsala University, Sweden.

## Abstract

The cytotoxic activity and cross-resistance pattern of the novel topoisomerase I inhibitor topotecan (Topo) were investigated in ten cell lines, representing different mechanisms of cytotoxic drug resistance, and in 218 fresh human tumour samples using the fluorometric microculture cytotoxicity assay (FMCA). Resistance to Topo in the cell lines was associated with expression of the multidrug resistance-associated protein (MRP), whereas the cell lines with P-glycoprotein (P-gp), topoisomerase II and glutathione-associated resistance did not show decreased sensitivity to the drug. Topo was more active in haematological than in solid tumour samples, but substantial activity was observed in carcinomas of the ovary and breast, sarcoma and childhood solid tumours. Cross-resistance to standard drugs representing different mechanisms of action was generally low in patient cells. The effect of Topo was better after longer exposure, but this time-dependent effect was largely abolished when adjustment for in vitro exposure was made. Topo showed activity both in proliferative and non-proliferative cell systems. The results indicate that Topo is insensitive to major mechanisms of resistance except for MRP. Proliferation does not seem to be necessary for the effect of Topo, and no superiority for protracted dosing schedules was observed. The results also suggest that, for example, leukaemias, lymphomas, sarcomas and childhood solid tumours may be suitable targets for future phase II trials.


British Journal of Cancer (1997) 76(2), 211–219
© 1997 Cancer Research Campaign

### Cytotoxic activity of topotecan in human tumour cell lines and primary cultures of human tumour cells from patients

E Jonsson[1], H Fridborg[1], K Csóka[1], S Dhar[1], C Sundström[2], P Nygren[3] and R Larsson[1]

[1]Division of Clinical Pharmacology, Departments of [2]Pathology and [3]Oncology, University Hospital, Uppsala University, S-751 85 Uppsala, Sweden

**Summary** The cytotoxic activity and cross-resistance pattern of the novel topoisomerase I inhibitor topotecan (Topo) were investigated in ten cell lines, representing different mechanisms of cytotoxic drug resistance, and in 218 fresh human tumour samples using the fluorometric microculture cytotoxicity assay (FMCA). Resistance to Topo in the cell lines was associated with expression of the multidrug resistance-associated protein (MRP), whereas the cell lines with P-glycoprotein (P-gp), topoisomerase II and glutathione-associated resistance did not show decreased sensitivity to the drug. Topo was more active in haematological than in solid tumour samples, but substantial activity was observed in carcinomas of the ovary and breast, sarcoma and childhood solid tumours. Cross-resistance to standard drugs representing different mechanisms of action was generally low in patient cells. The effect of Topo was better after longer exposure, but this time-dependent effect was largely abolished when adjustment for in vitro exposure was made. Topo showed activity both in proliferative and non-proliferative cell systems. The results indicate that Topo is insensitive to major mechanisms of resistance except for MRP. Proliferation does not seem to be necessary for the effect of Topo, and no superiority for protracted dosing schedules was observed. The results also suggest that, for example, leukaemias, lymphomas, sarcomas and childhood solid tumours may be suitable targets for future phase II trials.

**Keywords:** topotecan; cytotoxicity assay; human tumour cell; drug resistance

Topotecan (Topo) is a semisynthetic analogue of camptothecin, an alkaloid isolated from certain plant species such as *Camptotheca acuminata*. Camptothecin was found to be active against tumour cells in the National Cancer Institute (NCI) drug screening programme during the 1960s. Although its supposed mechanism of action was not clarified until the late 1980s, it was introduced into clinical trials during the 1970s. However, because of unexpected toxicity and limited activity in phase II trials further development of this group of drugs was halted until the more water-soluble and less toxic camptothecin analogues, among them Topo, were synthesized (Takimoto and Arbuck, 1996).

Topo is the first cytotoxic drug to be considered for approval for clinical use with the enzyme topoisomerase I as the probable target. At physiological pH the lactone form of Topo is in equilibrium with the open ring form, but only the former is active (Wall et al, 1992). Topoisomerase I is a nuclear enzyme present in all eucaryotic cell types, and is involved in DNA replication and repair. The enzyme unwinds the supercoiled double-stranded DNA by temporarily binding to, and cleaving, one of the strands. In the presence of Topo, the complex of cleaved DNA with topoisomerase I is stabilized, inhibiting religation (Takimoto and Arbuck, 1996). The drug-induced single-strand breaks are reversible, but as the replication goes on and the moving replication fork interacts with the drug–enzyme–DNA complex, double-strand breaks result that are thought to be responsible for the cytotoxic effects of Topo (Holm et al, 1989). Inhibition of DNA replication with, e.g. a DNA polymerase inhibitor that blocks double-strand break production reduces Topo cytotoxicity (Holm et al, 1989). Topo is therefore considered to be an S phase-specific drug, producing preferential toxicity to proliferating cells.

*Received 29 October 1996*
*Revised 13 January 1997*
*Accepted 17 January 1997*

*Correspondence to:* E Jonsson, Division of Clinical Pharmacology, Uppsala Akademiska Hospital, S-751 85 Uppsala, Sweden

Topo has shown cytotoxic activity against a broad range of cell types in vitro. Specific activity has been seen against colorectal, breast, non-small-cell lung (NSCLC), ovarian and renal cancers (Burris et al, 1992) and acute lymphocytic leukaemia (Uckun et al, 1995) in a clonogenic assay. In mice bearing human tumour xenografts Topo showed activity against rhabdomyosarcoma (Houghton et al, 1995) and central nervous system tumours (Friedman et al, 1994).

Topo has shown clinical activity in many tumour types, for example ovarian carcinoma (Gore et al, 1996), NSCLC (Perez-Solar et al, 1996) and leukaemia (Kantarjian et al, 1993; Rowinsky et al, 1994).

The mechanisms possibly responsible for resistance against Topo have been studied in various cell lines in vitro. The results from these studies are not conclusive but have indicated that resistance may be mediated by lowered levels and decreased sensitivity of the target enzyme (topoisomerase I) (Tan et al, 1989; Eng et al, 1990; Sorensen et al, 1995) and decreased drug accumulation unrelated to known mechanisms (Yang et al, 1995). Resistance may also, at least partly, be explained by sensitivity to P-glycoprotein 170 (P-gp)-mediated transport, although to a considerably lesser extent than known P-gp substrates (Hendricks et al, 1992; Mattern et al, 1993). An association between the heat shock protein hsp70 and decreased Topo sensitivity has been found in a murine fibrosarcoma cell line (Sliutz et al, 1996).

As a complement to ongoing clinical studies the present study was undertaken to characterize Topo in vitro with respect to tumour-type specificity, cross-resistance, schedule-dependency and possible mechanisms of resistance. This was carried out in a panel of human tumour cell lines and primary cultures of fresh human tumour cells from a broad spectrum of diagnoses and by use of the fluorometric microculture cytotoxicity assay (FMCA).

### MATERIALS AND METHODS

### Cell lines

To evaluate the resistance pattern of Topo, a human cell line panel of four sensitive parental cell lines, five drug resistant sublines, representing different mechanisms of resistance, and one cell line with primary resistance was used. The cell line panel has been described in detail previously (Dhar et al, 1996), and the basic information on each cell line together with references are given in Table 1. The cell line RPMI 8226/Dox40 shows the classical MDR phenotype with overexpression of 170 (P-gp). RPMI 8226/LR-5 shows a resistance proposed to be associated with increased levels of glutathione (GSH), whereas the resistance of U-937-Vcr is proposed to be tubulin associated. The H69AR cell line expresses a multidrug-resistant (MDR) phenotype proposed to be mediated by a multidrug resistance-associated protein (MRP) and the CEM/VM-1 expresses an atypical MDR, which is associated with altered topoisomerase II (topoII) activity. The exact mechanism of resistance for the primary resistant ACHN cell line is not known and may be multifactorial. The resistance patterns of the cell lines were routinely confirmed in control experiments.

### Immunocytochemistry

P-gp and MRP staining was performed on cytocentrifuge cell preparations with the monoclonal antibodies C219 and QCRL-1 (Centocor, Malvern, PA, USA) fixed in acetone and 70% methanol respectively, essentially as previously described (Cordell et al, 1984). Briefly, the specimens were incubated at a 1:10 dilution for 2 h at room temperature, followed by washing and application of a secondary rabbit anti-mouse antibody (Dako, Copenhagen, Denmark) for 30 min. After washing, a soluble complex of alkaline phosphatase and a mouse monoclonal anti-alkaline phosphatase (APAAP, Dako) was added for 30 min. The slides were developed using 10 mg ml$^{-1}$ Fast red (Sigma, St Louis, MO, USA) dissolved in a 0.5 M Tris buffer containing 2 mg ml$^{-1}$ Naphtol-As-Mx-phosphate and 2.4 mg ml$^{-1}$ levamisole (Sigma). The specimens were counterstained with Mayers haematoxylin and mounted. The fraction of tumour cells expressing positive staining was estimated after inspection of 100 cells on each slide.

### Patient samples

A total of 218 patient tumour samples from the different diagnoses, detailed in Table 2, were used to determine the activity of Topo and, for comparison, six other cytotoxic drugs, as detailed below, chosen to represent different mechanistic classes. However, because of a limited number of cells, all drugs could not be tested in all samples. Thirty solid and 20 haematological tumour samples and five preparations of normal peripheral blood mononuclear cells (PBMCs) from healthy blood donors were used to determine the dose–response relationship for Topo.

The tumour samples were obtained by bone marrow/peripheral blood sampling, routine surgery or diagnostic biopsy, and this sampling was approved by the local ethics committee at the Uppsala University Hospital. Leukaemic cells and PBMCs were isolated from bone marrow or peripheral blood by 1.077 g ml$^{-1}$ Ficoll–Paque (Kabi-Pharmacia, Uppsala, Sweden) density-gradient centrifugation (Larsson et al, 1992). Tumour tissue from solid tumour samples was minced into small pieces and tumour cells were then isolated by collagenase dispersion followed by Percoll (Kabi-Pharmacia) density-gradient centrifugation (Csoka et al, 1994). Cell viability was determined by trypan blue exclusion test and the proportion of tumour cells in the preparation was judged by inspection of May–Grünwald–Giemsa-stained cytospin preparations by a cytopathologist. In some cases, cells were cryopreserved in a culture medium containing 10% dimethylsulphoxide (DMSO; Sigma) and 50% inactivated fetal calf serum (FCS; HyClone, Cramlington, UK) by initial freezing for 24 h at –70°C, followed by storage in liquid nitrogen or at –150°C. Cryopreservation in this way does not affect drug sensitivity (Nygren et al, 1992).

### Reagents and drugs

Fluorescein diacetate (FDA; Sigma) was dissolved in DMSO and kept frozen (–20°C) as a stock solution protected from light. A complete medium consisting of culture medium RPMI-1640 (HyClone) supplemented with 10% inactivated FCS, 2 mM glutamine, 50 μg ml$^{-1}$ streptomycin and 60 μg ml$^{-1}$ penicillin was used throughout for both cell lines and patient samples.

Both in the cell lines and in the patient samples the activity of Topo and the standard drugs cisplatin, cytarabine, doxorubicin, etoposide, melphalan and taxol was determined. Additionally, camptothecin was tested in the cell lines. In the cell line panel all drugs were tested at five different drug concentrations, obtained by tenfold serial dilution from the maximum 100 μg ml$^{-1}$. In the patient samples, the concentrations chosen for the comparisons

**Table 1** Resistance factors (Rf) for Topo in cell lines. The resistance factor was defined as the $IC_{50}$ in the subline divided by that in its parental cell line

| Parental cell line | Resistant subline | Cell type | Selecting agent | Resistance mechanism | References | $R_f$ for Topo |
|---|---|---|---|---|---|---|
| CCRF-CEM | CEM/VM-1 | Leukaemia | Teniposide | TopoII-associated MDR | Danks et al (1988) | 1.1 |
| NCI-H69 | H69AR | SCLC | Doxorubicin | MRP-associated MDR | Cole et al (1992) | 76.9 |
| RPMI 8226/S | 8226/Dox40 | Myeloma | Doxorubicin | Pgp-associated MDR | Dalton et al (1986) | 1.1 |
| RPMI 8226/S | 8226/LR-5 | Myeloma | Melphalan | GSH-associated MDR | Mulcahy et al (1994) | 0.68 |
| U-937 GTB | U-937-Vcr | Lymphoma | Vincristin | Tubulin-associated MDR | Botling et al (1994) | 1.1 |
| ACHN | | Renal | | Primary resistant | Nygren and Larsson (1990) | |

SCLC, small-cell lung cancer; TopoII, topoisomerase II; MRP, multidrug-resistance protein; P-gp, P-glycoprotein 170; GSH, glutathione.

 © Cancer Research Campaign 1997

were the empirically derived cut-off concentrations (EDCCs), defined as the concentration that produces a significant scatter of survival index (SI) values among haematological tumours. This concentration was used to optimize the conditions for differentiating between sensitive and resistant tumour cell samples. The concentrations 1 μg $ml^{-1}$ and 0.5 μg $ml^{-1}$ were chosen for taxol and Topo respectively and the EDCCs for the other drugs have been described previously (Larsson et al, 1992). To determine the dose–response relationship for Topo in patient samples, five different drug concentrations were used, obtained by a fivefold serial dilution of the drug from 12.5 to 0.02 μg $ml^{-1}$.

Topo (SmithKline Beecham, King of Prussia, PA, USA) was dissolved and diluted in sterile water, and camptothecin (Sigma) was dissolved in methanol–chloroform and diluted further in phosphate-buffered saline (PBS; HyClone). The other drugs were obtained from commercial sources and were dissolved according to guidelines from the manufacturer and further diluted in PBS or sterile water.

Ninety-six-well microtitre plates (Nunc, Roskilde, Denmark) were prepared with 20 μl per well of drug solution at ten times the desired concentration, with the aid of a programmable pipetting robot (Propette, Perkin Elmer, Norwalk, CT, USA). The plates were stored frozen at –70°C for up to 2 months until further use. Under these conditions, no apparent change in drug activity was observed (Larsson et al, 1992).

### The fluorometric microculture cytotoxicity assay procedure

The fluorometric microculture cytotoxicity assay (FMCA) is based on measurement of fluorescence generated from hydrolysis of FDA to fluorescein by cells with intact plasma membranes and has been described in detail previously (Larsson et al, 1992). Briefly, the cells were resuspended in complete medium, and 180-μl cell suspension was seeded into the wells of 96-well experimental microtitre plates prepared with drugs as described. Each drug and concentration was tested in triplicate. Six wells, with cells but without drugs, served as control and six wells with only culture medium as blank. The plates were incubated at 37°C for 72 h, followed by aspiration of the medium, one wash in PBS and addition of 100 μl per well of FDA dissolved in PBS (10 μg $ml^{-1}$). The plates were incubated for 45 min and the generated fluorescence from each well was measured at 538 nm in a 96-well scanning fluorometer (Fluoroscan II, Labsystems Oy, Helsinki, Finland). The fluorescence is proportional to the number of viable cells in the well.

The stability of the drugs over the 72 h incubation at 37°C was investigated by a bioassay. Plates prepared with the different drugs were preincubated with 100 μl of medium per well for different time periods, ranging from 0 to 72 h at 37°C before cell suspension (RPMI 8226/S) was added. The activity of the drugs after different preincubation times was evaluated, by comparing the SI values resulting after 72 h incubation and measurement with FMCA as described above.

Quality criteria for a successful analysis included a fluorescence signal in the control wells of more than five times mean blank value, a mean coefficient of variation (CV) in the control wells of less than 30% and more than 70% tumour cells in the cell preparation before incubation.

**Table 2** Mean survival index (SI) and response rate to Topo at 0.5 μg $ml^{-1}$ for different diagnoses. Response rate was defined as the fraction of the samples with SI below the median SI (48%) for cell samples included

| Diagnosis | Mean SI (SD, %) | Response rate (%) | *n* |
|---|---|---|---|
| ALL | 49 (26) | 53 | 36 |
| AML | 45 (24) | 55 | 43 |
| CLL | 20 (20) | 94 | 16 |
| CML | 40 (26) | 63 | 6 |
| NHL | 26 (19) | 94 | 16 |
| Breast cancer | 68 (23) | 38 | 8 |
| Carcinoid | 109 (6) | 0 | 4 |
| Colorectal cancer | 93 (22) | 9 | 11 |
| NSCLC | 69 (29) | 19 | 21 |
| Ovarian carcinoma | 58 (29) | 40 | 25 |
| Sarcoma | 58 (23) | 50 | 6 |
| Childhood solid tumours | 61 (40) | 40 | 5 |
| Assorted tumours[a] | 66 (36) | 43 | 21 |

ALL, acute lymphocytic leukaemia; AML, acute myelocytic leukaemia; CLL, chronic lymphocytic leukaemia; CML, chronic myelocytic leukaemia; NHL, non Hodgkin's lymphoma; NSCLC, non-small-cell lung cancer. [a]The assorted tumour group includes: adenocortical cancer (three), bladder cancer (three), renal cell cancer (three), malignant histocytosis (two), small-cell lung cancer (two), adenocarcinoma (one), choriocarcinoma (one), parotis cancer (one), phaeochromocytoma (one), malignant melanoma (one), malignant mesothelioma (one), myeloma (one) and testicular tumour (one).

### Quantification of FMCA results

Cell survival is presented as survival index (SI), defined as the fluorescence in experimental wells as a per cent of that in control wells, with blank values subtracted. The $IC_{50}$ was defined as the Topo concentration giving a SI of 50%.

For the cell lines, the $IC_{50}$ values were evaluated for each individual cell line and drug with custom-made computer software. A Delta value was calculated as the logarithm of the $IC_{50}$ of the individual cell line minus the mean of all ten log $IC_{50}$ values (Boyd and Paul, 1995). The resistance factor for Topo in each subline was defined as the $IC_{50}$ of the resistant subline divided by the $IC_{50}$ of its sensitive parental cell line.

The $IC_{50}$ values for the haematological and solid patient samples respectively were determined graphically from the mean dose–response curves. Response rate to Topo was defined as the fraction of samples having a SI below the median SI at 0.5 μg $ml^{-1}$ for all samples investigated. The relative effect of a drug on solid and haematological tumours was indicated by the S/H ratio, defined as the ratio between the total response rates for the solid and the haematological samples.

### Dependency of Topo activity on dosing schedule and cell cycle phase

To evaluate the schedule dependency of Topo activity, cells were exposed to the same AUC (area under the concentration–time curve) of Topo but during different exposure times. CCRF-CEM cells and CLL patient cells were used. The cells were exposed to different Topo concentrations for 8 h, followed by washing with PBS, addition of new culture medium and continuation of incubation up to 72 h, before performing FMCA. In parallel, cells were exposed for the doubled concentrations for 4 h, and for four times the original concentrations for 2 h, before washing the Topo solution away.

© Cancer Research Campaign 1997

**Figure 1** Effect of Topo in the ten different cell lines as individual dose–response curves for each cell line (**A**) and expressed as Delta (**B**). Delta for a cell line was defined as the log $IC_{50}$ for the cell line minus the mean of the log $IC_{50}$s of all ten cell lines. A deflection to the right and left indicates lower and higher sensitivity than the mean respectively. **C** and **D** show the effect of doxorubicin and camptothecin respectively in the cell line panel. The mean log $IC_{50}$ was for Topo –1.23, for doxorubicin –0.25 and for camptothecin –0.09

To examine if the cells were proliferating during the 72 h incubation in the 96-well plates, bromodeoxyuridine (BrdU) incorporation into cellular DNA was determined with an ELISA kit from Boehringer Mannheim (Mannheim, Germany) essentially according to the protocol from the manufacturer. Briefly, CCRF/CEM cells and CLL patient cells seeded into 96-well plates were incubated for 72 h together with the pyrimidine analogue BrdU, which incorporates into DNA of proliferating cells. Then the cells were fixed and an antibody against BrdU was added. The immune complex was detected by a substrate reaction using tetramethylbenzidine (TMB), giving a product that was measured in a spectrophotometric microplate reader (Dynatech Laboratories, Billingshurst, UK).

### RESULTS

The dose–response curves for Topo in the individual cell lines are shown in Figure 1A. The SCLC cell line NCI-H69 and its subline H69AR were the most resistant to Topo, together with the renal carcinoma cell line ACHN. The cell lines of haematological origin were more and equally sensitive, except for the myeloma cell line RPMI 8226/S with its sublines 8226/Dox40 and 8226/LR-5, which were of intermediate sensitivity. The experiments were performed twice with similar results and the mean value was used. The greatest difference in sensitivity between a cell line and its subline was seen for NCI-H69 and its MRP-expressing resistant subline H69AR, where the resistance factor was 77 (Table 1). Almost no decreased sensitivity compared with parental cell lines was observed in the sublines with topoII-, P-gp- and tubulin-associated MDR, whereas the 8226/LR-5 subline with GSH-associated MDR showed a slightly increased sensitivity.

Figure 1B–D shows the Delta values, i.e. the deviation of log $IC_{50}$ for each cell line from the mean log $IC_{50}$ of the cell line panel, for Topo (1B) and for comparison for doxorubicin (1C) and camptothecin (1D). Camptothecin showed a sensitivity pattern similar to that of Topo, the solid tumour- and myeloma cell lines being more resistant than the haematological ones. Differences between parental cell line and resistant subline were seen only for NCI-H69AR and its MRP-expressing subline H69AR, and for RPMI 8226/S and its subline 8226/LR-5 with GSH-mediated resistance.

 © Cancer Research Campaign 1997

**Figure 2** Dose–response relationship for Topo in (**A**) solid tumours, (**B**) haematological tumours and (**C**) normal peripheral blood mononuclear cells (PBMCs). Five PBMC preparations, 20 haematological and 30 solid samples were tested. The bars indicate standard errors of the mean

Another pattern was seen for doxorubicin, which was very active against RPMI 8226/S, and which was influenced by all the resistance mechanisms, all the sublines being less sensitive than their parental cell lines.

When examining the immunocytochemical staining of the cell lines, it was found that the only cell line expressing MRP was H69-AR, with 90% of the cells showing positive staining. P-gp was highly expressed in the doxorubicin-selected cell line 8226/Dox40 (80% positively stained), but also to a minor extent in the lymphoma cell lines U937 GTB (50%) and U937-Vcr (60%). Preliminary data indicate that the MRP inhibitor genistein (Verovski et al, 1996) is able to increase the sensitivity for Topo for the MRP-expressing cell line H69-AR (unpublished data).

The haematological patient samples and the normal PBMCs showed quite similar Topo sensitivity, with $IC_{50}$ values of 66 ng $ml^{-1}$ and 48 ng $ml^{-1}$ respectively, and were more sensitive than the solid samples in which no $IC_{50}$ value was obtained (Figure 2). At the highest Topo concentration the SI was 59% in the solid and 21% in the haematological samples.

In Table 1 the response rates to Topo at 0.5 μg $ml^{-1}$ for the patient samples are listed according to diagnoses. The haematological tumours showed the highest response rates: 53–94% in the lymphocytic leukaemias, 55–63% in the myelocytic leukaemias and 94% in the non-Hodgkin's lymphomas. Among the solid tumours, the highest response rates were observed in sarcomas (50%), childhood solid tumours (40%), ovarian carcinoma (40%) and breast cancer (38%). In the assorted tumour group the overall response rate was 43%, with responses seen in small-cell lung cancer (2 out of 2), choriocarcinoma (1 out of 1), malignant mesothelioma (1 out of 1), bladder cancer (1 out of 3), adrenocortical carcinoma (1 out of 3) and in the haematological tumours (3 out of 3).

The relative effect of the drugs in solid, compared with haematological, tumour samples expressed as the S/H ratio is shown in Figure 3. Of the six different drugs compared, taxol showed the greatest relative effect on solid tumours (S/H ratio 1.06), followed by cisplatin (0.80), etoposide (0.48) and Topo (0.45). Cytarabine was the drug with the most pronounced haematological tumour activity pattern (S/H ratio 0.12).

The correlation coefficients in haematological patient samples between the SI of Topo and the SI of each of the standard cytotoxic drugs at their EDCCs were compared pairwise and listed in Table 3. Topo and taxol showed low correlations with the standard drugs, with coefficients of correlation under 0.3. The coefficients of correlation between the other standard drugs were higher and varied between 0.31 and 0.68.

When comparing the effect of Topo after different exposure times, the longer exposure times gave a better effect than the shorter ones, both in CCRF/CEM and in CLL patient cells (Figure 4A and C). However, when the test concentrations were compensated for the greater AUC resulting from a longer exposure time, the differences between the different dosing schedules were diminishing, leaving only a very small tendency towards a preference for prolonged exposure (Figure 4B and D). As Topo was shown to be stable under 72 h FMCA conditions (data not shown), the AUC was calculated as the Topo concentration multiplied by the exposure time.

The proliferative activity of the CCRF/CEM cells and the CLL patient cells in Figure 4, expressed as absorbance at 450 nm per 10 000 seeded cells after a performed proliferation ELISA is shown in Figure 5. The CLL patient cells show no proliferation, whereas the CCRF/CEM cells show a substantial proliferation, which is inhibited in a dose-dependent fashion by Topo.

© Cancer Research Campaign 1997

**Figure 3** S/H ratios, defined as the ratio between the response rates of solid and haematological samples, for different drugs at their empirically derived cut-off concentrations (EDCCs). The number of samples investigated for Topo was as defined in Table 2, whereas the number of samples for the standard drugs ranged 96–113 for haematological and 43–89 for solid tumours

**Figure 5** Proliferation of CEM/S cells (-o-) and primary cultures of CLL cells from patients (-◆-) for 72 h under FMCA conditions in the presence of different concentrations of Topo. The proliferation is expressed as the absorbance at 450 nm per 10 000 cells after the ELISA procedure described in Materials and methods

### DISCUSSION

The mechanisms of resistance to Topo are not completely understood. Some preclinical cell line data indicate that Topo appears to be part of the P-gp-mediated multidrug-resistance phenotype, but the effect of P-gp-mediated transport on Topo accumulation in cells is substantially smaller compared with classical P-gp substrates (Hendricks et al, 1992; Mattern et al, 1993). According to our findings, the P-gp-mediated resistance does not seem to influence the effect of Topo to any greater extent. However, the MRP-expressing cell line H69AR was found to be 77-fold more

**Figure 4** Schedule dependency of the effect of Topo. Dose–response relationship for Topo in CCRF/CEM cells (**A** and **B**) and CLL cells (**C** and **D**) when exposing the cells for Topo for 2 (-o-), 4 (-⊞-) and 8 (-▲-) h respectively. The exposure expressed as Topo concentration (**A** and **C**) and AUC (**B** and **D**)

 © Cancer Research Campaign 1997

**Table 3** Correlation between the SI values in haematological samples for the indicated drugs at their empirically derived cut-off concentrations (EDCCs). Each correlation is based on 77–112 data points

| | Cytarabine (anti-metabolite) | Cisplatin (platinum compound) | Doxorubicin (DNA intercalator) | Etoposide (topo II inhibitor) | Melphalan (alkylating agent) | Taxol (tubulin active) |
|---|---|---|---|---|---|---|
| Cisplatin | 0.47 | | | | | |
| Doxorubicin | 0.51 | 0.43 | | | | |
| Etoposide | 0.31 | 0.36 | 0.52 | | | |
| Melphalan | 0.44 | 0.45 | 0.66 | 0.68 | | |
| Taxol | 0.26 | –0.03 | 0.22 | 0.21 | 0.05 | |
| Topotecan | 0.23 | 0.02 | 0.22 | 0.10 | 0.20 | 0.28 |

resistant to Topo than the parental cell line, suggesting that MRP expression may lead to Topo resistance. The pattern of MRP, but not that of P-gp, expression also closely paralleled that of the resistance factors. The preliminary data pointing to a possibility of making the resistant cell line H69AR more sensitive to Topo by adding the MRP inhibitor genistein further indicate a role of MRP in Topo resistance. A high degree of cross-resistance to Topo has been observed by Yang et al (1995) in a mitoxantrone-selected, P-gp-negative cell line with MRP expression. However, as its parental cell line showed similar expression of MRP, the decreased Topo accumulation and cytotoxic activity in the resistant cell line was suggested to be mediated by an unknown mechanism (Yang et al, 1995). It is not known whether the H69AR cells also have similar mechanisms operating in addition to MRP.

Investigating the relationship between topoisomerase I and MRP expression and clinical response to Topo therapy would be a highly interesting subject for future studies aiming at elucidating the clinically relevant mechanisms of resistance.

When comparing the sensitivity pattern of Topo in the cell line panel with that of its parent compound camptothecin, a similarity was seen. Topo seems to share the MRP-associated resistance with its parent compound. On the other hand, doxorubicin showed an entirely different sensitivity pattern, and was influenced by all the resistance mechanisms represented in the panel. Comparison of the sensitivity pattern of different drugs in the cell line panel can be used to detect mechanistic similarity between compounds (Dhar et al, 1996). When the drugs were compared in the patient samples, very low levels of cross-reactivity were observed between Topo and standard drugs representing the major types of mechanisms of action. The standard drugs correlated substantially more with each other, and it has been shown previously that drugs from the same mechanistic classes show even higher correlations (correlation coefficients > 0.70) (Jonsson et al, 1996). This indicates a potential role of Topo in combination therapy.

The activity of Topo has previously been investigated in clonogenic assays of freshly explanted specimens from patients with various solid tumours (Burris et al, 1992). In that study, carcinomas of the kidney, lung and breast were found to be most sensitive to Topo. Ovarian carcinoma was found to be less sensitive, giving responses of the same magnitude as, for example, colorectal cancer. These results contrast to the present results in which renal cell carcinoma and colorectal tumours were largely insensitive whereas ovarian carcinoma was sensitive to Topo. The reason for the apparent discrepancy may at least be related partly to the different end points used, i.e. proliferation of clonogenic cells used in the clonogenic assay and cell death used in the FMCA. If prediction of tumour regression in vivo is sought, cell kill may theoretically be a more appropriate and robust end point to measure. In practice, the correlation with known clinical response pattern is the most important argument for the validity of any end point or assay type.

Clinical studies of the anti-tumour activity of Topo have been performed in a variety of tumor types. In ovarian carcinoma, Topo has shown promising phase II activity with reported response rates of around 20% in relapsed patients (Gore et al, 1996). Activity of Topo has also been reported for NSCLC (Perez-Soler et al, 1996), SCLC (Ardizzoni et al, 1994; Shiller et al, 1994), soft tissue sarcoma (Eisenhauer et al, 1994) and breast cancer (Chang et al, 1995). In renal cell cancer (Ilson et al, 1993; Law et al, 1994) and colorectal cancer (Sugarman et al, 1994) the drug appears inactive. Little phase II experience has been reported for haematological tumours, although responses in phase I trials have been observed in patients with relapsed or refractory acute leukaemia (Kantarjian et al, 1993; Rowinsky et al, 1994).

The present 'in vitro phase II' results using cell kill as end point appears therefore to be more in accordance with the reported clinical activity than the clonogenic assay. As for the majority of currently used chemotherapeutic drugs, Topo showed a high degree of differential activity against haematological tumours in the present study. The present results indicate that in addition to various haematological tumours sarcomas, childhood solid tumours and breast cancer may be suitable targets for clinical phase II studies.

The in vitro effect of Topo has previously been shown to be dependent on the exposure time, with longer incubations being more active than shorter ones (Burris et al, 1992; Cheng et al, 1994; Uckun et al, 1995). However, the drug effects were not adjusted for the total exposures precluding any firm conclusions about superiority for protracted scheduling (true 'time dependency'). In the present study, the apparent time dependency of the effect on both CCRF-CEM and CLL patient cells was largely abolished when adjustments for in vitro exposure was made. Thus, for the different exposure times tested in the present study, the drug effects were best explained by the area under the concentration–time curve (AUC). Furthermore, Topo was clearly highly active against patient CLL cells showing no evidence of DNA synthesis during the 72-h assay period, strongly arguing against a requirement for S-phase progression, at least for this cell type.

In vivo, however, the question of dosing schedule dependency is more complicated and involves both pharmacokinetic factors and the toxic effects on normal host cells. A single optimal schedule could not be determined from the early preclinical in vivo studies (Dancey and Eisenhauer, 1996), although support for protracted schedules was obtained from some tumour model systems (Houghton et al, 1992). Nevertheless, the most commonly used clinical phase II schedule, a daily × five schedule, has been

© Cancer Research Campaign 1997

compared with more protracted schedules without any apparent advantage of the latter (Dancey and Eisenhauer, 1996). This clinical experience and the present results may thus suggest that the optimal schedule for Topo is yet to be defined for different tumour types and that additional short-term schedules might be explored. In this context it should be noted that, also from a theoretical point of view, AUC vs time dependency for drugs with short half-lives may vary depending on the sensitivity of the tumour model system used, irrespective of cell cycle phase specificity (Karlsson et al, 1996).

We have previously shown that the FMCA can detect tumour-type specific activity retrospectively for a series of standard drugs (Nygren et al, 1994) and prospectively for early phase I–II drugs such as 2-chlorodeoxyadenosine (Larsson et al, 1994), gemcitabine (Csoka et al, 1995) and taxol (Nygren et al, 1995). The use of a human cell line panel can contribute with information on resistance mechanisms and on mechanistic similarity to other standard and investigational agents (Dhar et al, 1996). The parallel or sequential application of these model systems may provide important initial information on selectivity and similarity of novel anticancer drugs.

### ACKNOWLEDGEMENTS

This study was supported by grants from the Swedish Cancer Society and the Lions Cancer Foundation. The skilful technical assistance of Ms Charlotta Sandberg, Mrs Carina Alvfors and Ms Christina Hedin and the cytopathological expertise provided by Dr Jörgen Kristensen and Dr Manuel de la Torre are gratefully acknowledged.

### REFERENCES

Ardizzoni A, Hansen H and Dombernowsky P (1994) Phase II study of topotecan in pretreated small cell lung cancer (SCLC). *Proc Annu Meet Am Soc Clin Oncol* **13**: A1116

Botling J, Liminga G, Larsson R, Nygren P and Nilsson K (1994) Development of vincristine resistance and increased sensitivity to cyclosporin A and verapamil in the human U-937 lymphoma cell line without overexpression of the 170 kDa p-glycoprotein. *Int J Cancer* **58**: 269–274

Boyd M and Paull K (1995) Some practical considerations and applications of the national cancer institute in vitro anticancer drug discovery screen. *Drug Develop Res* **34**: 91–109

Burris H, Hanauske A, Johnsson R, Marshall M, Kuhn J, Hilsenbeck SG and von Hoff D (1992) Activity of topotecan, a new topoisomerase I inhibitor, against human tumor colony-forming units in vitro. *J Natl Cancer Inst* **84**: 1816–1819

Chang A, Garrow G, Boros L, Asbury R, Pandya K and Keng P (1995) Clinical and laboratory studies of topotecan in breast cancer. *Proc Annu Meet Am Soc Clin Oncol* **14**: A118

Cheng M, Chatterjee S and Berger N (1994) Schedule-dependent cytotoxicity of topotecan alone and in combination chemotherapy regimens. *Oncol Res* **6**: 269–279

Cole S, Bhardwaj G, Gerlach J, Mackie J, Grant C, Almquist K, Stewart A, Kurz E, Duncan A and Deeley R (1992) Overexpression of a transporter gene in a multidrug-resistant human lung cancer cell line. *Science* **258**: 1650–1654

Cordell J, Falini B and Erber W (1984) Immunoenzymatic labeling of monoclonal antibodies using immune complexes of alkaline phosphatase and monoclonal anti-alkaline phosphatase (APAAP complexes). *J Histochem Cytochem* **32**: 219–229

Csoka K, Larsson R, Tholander B, Gerdin E, De La Torre M and Nygren P (1994) Cytotoxic drug sensitiviy testing of tumor cells from patients with ovarian carcinoma using the fluorometric microculture cytotoxicity assay (FMCA). *Gynecol Oncol* **54**: 163–170

Csoka K, Liliemark J, Larsson R and Nygren P (1995) Evaluation of the cytotoxic activity of gemcitabine in primary cultures of tumor cells from patients with hematologic or solid tumors. *Semin Oncol* **22**: 47–53

Dalton W, Durie B, Alberts D, Gerlach J and Cress A (1986) Characterization of a new drug-resistant human myeloma cell line that expresses p-glycoprotein. *Cancer Res* **46**: 5125–5130

Dancey J and Eisenhauer E (1996) Current perspectives on camptothecins in cancer treatment. *Br J Cancer* **74**: 327–338

Danks M, Schmidt C, Cirtain M, Suttle D and Beck W (1988) Altered catalytic activity of and DNA cleavage by DNA topoisomerase II from human leukemic cells selected for resistance to VM-26. *Biochemistry* **27**: 8861–8869

Dhar S, Nygren P, Csoka K, Botling J, Nilsson K and Larsson R (1996) Anticancer drug characterization using human cell line panel representing defined types of drug resistance. *Br J Cancer* **74**: 888–896

Eisenhauer E, Wainman N, Boos G, Macdonald D and Bramwell V (1994) Phase II trials of topotecan in patients (pts) with malignant glioma and soft tissue sarcoma. *Proc Annu Meet Am Soc Clin Oncol* **13**: A488

Eng W, McCabe F, Tan K, Mattern M, Hofmann G, Woessner R, Hertzberg R and Johnsson R (1990) Development of a stable camptothecin-resistant subline of P388 leukemia with reduced topoisomerase I content. *Mol Pharmacol* **38**: 471–480

Friedman H, Houghton P, Schold S, Keir S and Bigner D (1994) Activity of 9-dimethylaminomethylcamptothecin against pediatric and adult central nervous system tumor xenografts. *Cancer Chemother Pharmacol* **34**: 171–174

Gore M, Bolis B, Creemers G, Despax R, Guastalla J, Lacave J, Mee D, Scarfone G, ten Bokkel Huinink W and van Belle S (1996) A phase II study of topotecan as second-line therapy given as five daily doses for advanced epithelial ovarian cancer. *9th NCI-EORTC Symposium on New Drugs in Cancer Therapy, Amsterdam*, A474.

Hendricks C, Rowinsky E, Grochow L, Donehower R and Kaufmann S (1992) Effect of p-glycoprotein expression on the accumulation and cytotoxicity of topotecan (SK&F 104864), a new camptothecin analogue. *Cancer Res* **52**: 2268–2278

Holm C, Covey J, Kerrigan D and Pommier Y (1989) Differential requirement of DNA replication for the cytotoxicity of DNA topoisomerase I and II inhibitors in chinese hamster DC3F cells. *Cancer Res* **49**: 6365–6368

Houghton P, Cheshire P, Myers L, Stewart C, Synold T and Houghton J (1992) Evaluation of 9-dimethylaminomethyl-10-hydroxycamptothecin against xenografts derived from adult and childhood solid tumors. *Cancer Chemother Pharmacol* **31**: 229–239

Houghton P, Cheshire P, Hallman J, Lutz L, Friedman H, Danks M and Houghton J (1995) Efficacy of topoisomerase I inhibitors, topotecan and irinotecan, administered at low dose levels in protracted schedules to mice bearing xenografts of human tumors. *Cancer Chemother Pharmacol* **36**: 393–403

Ilson D, Motzer R, O'Moore P, Nanus D and Bosl G (1993) A phase II trial of topotecan in advanced renal cell carcinoma. *Proc Annu Meet Am Soc Clin Oncol* **12**: A779

Jonsson B, Liminga G, Csoka K, Fridborg H, Dhar S, Nygren P and Larsson R (1996) Cytotoxic activity of Calcein acetoxymethyl ester (Calcein/AM) on primary cultures of human haematological and solid tumours. *Eur J Cancer* **32A**: 883–887

Kantarjian H, Beran M, Ellis A, Zwelling L, O'Brien S, Cazenave L, Koller C, Rios M, Plunkett W, Keating W and Estey E (1993) Phase I study of topotecan, a new topoisomerase inhibitor, in patients with refractory or relapsed acute leukemia. *Blood* **81**: 1146–1151

Karlsson M, Molnar V, Bergh J, Freijs A and Larsson R (1996) A general model for time-dissociated PK/PD relationships. Manuscript.

Larsson R, Kristensen J, Sandberg C and Nygren P (1992) Laboratory determination of chemotherapeutic drug resistance in tumor cells from patients with leukemia using a fluorometric microculture cytotoxicity assay (FMCA). *Int J Cancer* **50**: 177–185

Larsson R, Fridborg H, Liliemark J, Csoka K, Kristensen J, de la Torre M and Nygren P (1994) In vitro activity of 2-chlorodeoxyadenosine (CdA) in primary cultures of human haematological and solid tumours. *Eur J Cancer* **30A**: 1022–1026

Law T, Ilson D and Motzer R (1994) Phase II trial of topotecan in patients with advanced renal cell carcinoma. *Invest New Drugs* **12**: 143–145

Mattern M, Hofmann G, Polsky R, Funk L, McCabe F and Johnson R (1993) In vitro and in vivo effects of clinically important camptothecin analogues on multidrug-resistant cells. *Oncol Res* **5**: 467–474

Mulcahy R, Bailey H and Gipp J (1994) Up-regulation of gamma-glutamylcysteine synthetase activity in melphalan-resistant human multiple myeloma cells expressing increased glutathione levels. *Cancer Chemother Pharmacol* **34**: 67–71

Nygren P and Larsson R (1990) Verapamil and cyclosporin A sensitize human kidney tumor cells to vincristine in absence of membrane p-glycoprotein and without apparent changes in the cytoplasmic free $Ca^{2+}$ concentration. *Biosci Rep* **10**: 231–237

Nygren P, Kristensen J, Sundström C, Lönnerholm G, Kreuger A and Larsson R (1992) Feasibility of the fluorometric microculture cytotoxicity assay (FMCA)

© Cancer Research Campaign 1997

for cytotoxic drug sensitivity testing of tumor cells from patients with acute lymphoblastic leukemia. *Leukemia* **11**: 1121–1128

Nygren P, Fridborg H, Csoka K, Sundström C, De La Torre M, Kristensen J, Bergh J, Hagberg H, Glimelius B, Rastad J, Tholander B and Larsson R (1994) Detection of tumor-specific cytotoxic drug activity in vitro using the fluorometric microculture cytotoxicity assay and primary cultures of tumor cells from patients. *Int J Cancer* **56**: 715–720

Nygren P, Csoka K, Jonsson B, Fridborg H, Bergh J, Hagberg H, Glimelius B, Brodin O, Tholander B, Kreuger A, Lönnerholm G, Jakobsson Å, Olsen L, Kristenssen J and Larsson R (1995) The cytotoxic activity of Taxol in primary cultures of tumor cells from patients is partly mediated by Cremophor EL. *Br J Cancer* **71**: 478–481

Perez-Soler R, Fossella F, Glisson B, Lee J, Murphy W, Shin D, Kemp B, Lee J, Kane J, Robinson R, Lippman S, Kurie J, Huber M, Raber M and Hong W (1996) Phase II study of topotecan in patients with advanced non-small-cell lung cancer previously untreated with chemotherapy. *J Clin Oncol* **14**: 503–513

Rowinsky E, Adjei A, Donehower R, Gore S, Jones R, Burke P, Cheng Y, Grochow L and Kaufmann S (1994) Phase I and pharmacodynamic study of the topoisomerase I-inhibitor topotecan in patients with refractory acute leukemia. *J Clin Oncol* **12**: 2193–2203

Shiller J, Kim K and Johnson D (1994) Phase II study of topotecan in extensive stage small cell lung cancer. *Proc Annu Meet Am Soc Clin Oncol* **13**: A330

Sliutz G, Karlseder J, Tempfer C, Orel L, Holzer G and Simon M (1996) Drug resistance against gemcitabine and topotecan mediated by constitutive hsp70 overexpression in vitro: implication of quercetin as sensitiser in chemotherapy. *Br J Cancer* **74**: 172–177

Sorensen M, Sehested M and Jensen P (1995) Characterisation of a human small-cell lung cancer cell line resistant to the DNA topoisomerase I-directed drug topotecan. *Br J Cancer* **72**: 399–404

Sugarman S, Ajani J, Daugherty K, Winn R, Lanzotti V, Bearden J and Abbruzzese J (1994) A phase II trial of topotecan (TPT) for the treatment of advanced, measurable colorectal cancer. *Proc Annu Meet Am Soc Clin Oncol* **13**: A686

Takimoto C and Arbuck S (1996) The camptothecins. In *Cancer Chemotherapy and Biotherapy – Principles and Practice*, Chabner B and Longo D (eds), p. 463. Lippincott: Philadelphia

Tan K, Mattern M, Eng W, McCabe F and Johnson R (1989) Nonproductive rearrangement of DNA topoisomerase I and II genes: correlation with resistance to topoisomerase inhibitors. *J Natl Cancer Inst* **81**: 1732–1735

Uckun F, Stewart G, Reaman G, Chelstrom L, Jin J, Chandan-Langlie M, Waddick K, White J and Evans W (1995) In vitro and in vivo activity of topotecan against human B-lineage acute lymphoblastic leukemia cells. *Blood* **85**: 2817–2828

Verovski V, Van den Berge D, Delvaeye M, Scheper R, De Neve W and Storme G (1996) Low-level doxorubicin resistance in P-glycoprotein negative human pancreatic tumor PSN1/ADR cells implicates a brefeldin A-sensitive mechanism of drug extrusion. *Br J Cancer* **73**: 596–602

Wall J, Burris H, Von Hoff D, Rodriguez G, Kneuper-Hall R, Shaffer D, O'Rourke T, Brown T, Weiss G, Clark G, McVea S, Brown J, Johnson R, Friedman C, Smith B, Mann W and Kuhn J (1992) A phase I clinical and pharmacokinetic study of the topoisomerase I inhibitor topotecan (SK&F 104864) given as an intravenous bolus every 21 days. *Anti-Cancer Drugs* **3**: 337–345

Yang C, Horton J, Cowan K and Schneider E (1995) Cross-resistance to camptothecin analogues in a mitoxantrone-resistant human breast carcinoma cell line is not due to DNA topoisomerase I alterations. *Cancer Res* **55**: 4004–4009

© Cancer Research Campaign 1997